# Animal Ownership and Touching Enrich the Context of Social Contacts Relevant to the Spread of Human Infectious Diseases

**DOI:** 10.1371/journal.pone.0133461

**Published:** 2015-07-20

**Authors:** Yimer Wasihun Kifle, Nele Goeyvaerts, Kim Van Kerckhove, Lander Willem, Christel Faes, Herwig Leirs, Niel Hens, Philippe Beutels

**Affiliations:** 1 Center for Health Economics Research & Modeling of Infectious Diseases (CHERMID), Vaccine and Infectious Disease Institute, University of Antwerp, Antwerp, Belgium; 2 Interuniversity Institute for Biostatistics and statistical Bioinformatics (I-BioStat), Hasselt University, Diepenbeek, Belgium; 3 Department of Mathematics and Computer Science, University of Antwerp, Antwerp, Belgium; 4 Evolutionary Ecology Group, University of Antwerp, Antwerp, Belgium; 5 School of Public Health and Community Medicine, The University of New South Wales, Sydney, Australia; Centre de Physique Théorique, FRANCE

## Abstract

Many human infectious diseases originate from animals or are transmitted through animal vectors. We aimed to identify factors that are predictive of ownership and touching of animals, assess whether animal ownership influences social contact behavior, and estimate the probability of a major zoonotic outbreak should a transmissible influenza-like pathogen be present in animals, all in the setting of a densely populated European country. A diary-based social contact survey (n = 1768) was conducted in Flanders, Belgium, from September 2010 until February 2011. Many participants touched pets (46%), poultry (2%) or livestock (2%) on a randomly assigned day, and a large proportion of participants owned such animals (51%, 15% and 5%, respectively). Logistic regression models indicated that larger households are more likely to own an animal and, unsurprisingly, that animal owners are more likely to touch animals. We observed a significant effect of age on animal ownership and touching. The total number of social contacts during a randomly assigned day was modeled using weighted-negative binomial regression. Apart from age, household size and day type (weekend versus weekday and regular versus holiday period), animal ownership was positively associated with the total number of social contacts during the weekend. Assuming that animal ownership and/or touching are at-risk events, we demonstrate a method to estimate the outbreak potential of zoonoses. We show that in Belgium animal-human interactions involving young children (0–9 years) and adults (25–54 years) have the highest potential to cause a major zoonotic outbreak.

## Introduction

Many emerging and re-emerging infectious diseases have been transmitted to human populations via animals [1–4], creating a public health interest in human-animal interactions. This is of particular importance in low and middle-income countries where social, cultural and environmental circumstances may increase interspecies transmission, with the potential to start a new pandemic in humans. Nonetheless, also in high-income countries (HICs), animals play an important role in transmitting infectious diseases to humans such as hantavirus, rabies, tick-borne encephalitis and Lyme disease, though these have generally no potential to spread further between humans. Nevertheless in theory avian influenza could be transmitted from poultry in any type of country. In HICs, about half of the households own pets [5] and these companion animals may carry zoonotic infections [6]. In the current paper, we study both ownership and touching of animals, since they both represent important risk factors for zoonotic infectious disease transmission. We applied similar methodologies as in earlier large-scale surveys on social contact patterns [7] and extended these with human-animal interactions. We relate these human-animal interactions with social contact frequencies between humans, and demonstrate a method to estimate the associated risks of causing a major zoonotic outbreak, conditional on a flu-like pathogen being present in each of the animal groups.

## Methods

### Social contact survey

Social contact surveys have proven to be very useful to understand and model the spread of infectious diseases [7–12]. We conducted a diary-based social contact survey in the Flemish region of Belgium from September 2010 till February 2011. Participants were recruited by random digit dialing on mobile phones and landlines. Quota sampling by age, gender and region was applied in order to achieve a representative sample population. Only one person per household was included in the survey. Participants recorded their social contacts in a paper diary during one randomly assigned day and completed a background survey.

Two general characteristics were used to define inter-human contacts in this study, with the aim to capture direct contacts that are relevant for infectious disease transmission. First, a contact was recorded when a participant engaged in a direct conversation with someone else at most three meters away (e.g. phone or internet communications were excluded). Second, if a participant touched someone else (e.g. shaking hands), this was considered as a “physical” contact, even if not a word was spoken.

In addition to inter-human contacts, participants were requested to complete enquiries about human-animal interactions (For clarification see the sample questionnaire on **S1 Fig**).

Animal ownership was defined as having at least one live animal in the household in which the participant was spending the majority of his/her time. Animal touching was defined as touching at least one living animal on the assigned day, irrespective of whether that animal belonged to the household. After data collection, for clarity of presentation, we grouped animals into four classes: pets (cat, dog, fish), livestock (horse, sheep, pig, cow), poultry (chicken, turkey, pigeon) and “other”.

We used three diary types, adjusted to the age of the participants. For children (0–12 years), engagement in a direct conversation was extended to include babbling, laughing, crying or playing. These diaries were completed by a proxy (relatives, caregivers and teachers). The diaries for people of 13–60 years contained a section in which high numbers of contacts (henceforth referred to as “professional” contacts) could be reported. For the elderly (> 60 years), there were also instructions for proxies to help the participants.

Participants were reminded by phone to fill in the diary one day before the assigned day and followed up the day after. Physical samples were not collected as part of this study. The age-specific diaries were sent and returned via mail in a pre-stamped envelope. The data were single-entered in an electronic database and checked manually. The ethical committee of the Antwerp University Hospital approved the study protocol.

### Modeling human-animal interactions

In this section, we discuss relevant demographic and temporal factors, and methods to model the number of social contacts together with animal ownership and touching.

#### Demographic and temporal factors

The following demographic factors were considered when modeling animal ownership and touching and the number of social contacts between humans: age, gender, educational attainment, province and household size. We used six age categories based on Belgian schooling system: 0–5, 6–11, 12–17, 18–44, 45–64 and 65+ years. Further descriptions can be found in **S1 File**.

Additionally, some temporal characteristics were considered when modeling the occurrence of contacts. Based on the school holidays in Flanders, which include public holidays, the assigned date was categorized as a holiday or regular day. Weekdays (Monday-Friday) were also distinguished from weekend days (Saturday-Sunday).

#### Modeling animal ownership and touching

We used multiple logistic regression to model the probability of owning and touching animal(s). The total number of contacts, animal ownership, as well as all the demographic and temporal factors discussed above were included as possible predictors of animal touching, whereas only the socio-demographic factors were included as possible predictors of animal ownership.

We used stepwise backward model selection based on Akaike’s Information Criterion (AIC) [13]. A likelihood ratio test (at 5% level of significance) was used to check the overall significance of covariates in the model selected with AIC, excluding non-significant covariates to obtain the final model. Significant two-way interactions obtained from all possible pairs of predictors were retained (based on likelihood ratio test). Next, we also analyzed animal touching by specifying cat, dog, livestock and poultry ownership as possible predictors instead of animal ownership. Finally, using the same model building strategy, we also analyzed the odds of owning and touching cats, dogs and/or pets (cat, dog, fish).

#### Modeling number of contacts

The number of contacts was defined as the total number of contacts including professional contacts (physical and non-physical) reported by a participant during the assigned day. We used a weighted negative binomial regression model for the total number of contacts, which explicitly accounts for overdispersion [14, 15]. Post-stratification weights based on age and household size were used (from the 2000 Belgium census data) to estimate population-related quantities [7, 16]. These weights were constrained to a maximum of 3 to limit the influence of larger weights. The model selection criteria used in Section 2.2.2 were also applied here. We included demographic and temporal factors and animal ownership as possible predictors of the total number of contacts, together with significant two-way interactions (based on likelihood ratio tests). We also modeled the total number of contacts with cat, dog, livestock and poultry ownership separately. Finally, we used the same modeling approach for the number of physical contacts, which are considered as inter-human contacts with a high transmission potential.

#### Major outbreak probability of zoonoses with flu-like transmissibility, conditional on their presence in animal groups

We assumed that animal pathogens could infect humans through owning or touching infected pets, livestock and poultry. Some groups of people in contact with these infected animals are at higher risk of infection than others due to their age and socio-demographic background. We estimated the probability that a certain human-transmissible pathogen would cause a major outbreak in a specific age group (see S5 File for details about the method), conditional on the pathogen being present in the animals, and being able to transmit to humans with a frequency that is proportional to the frequency of touching or owning these animals. By way of example and without loss of generalisability we assumed a basic reproduction number of 1.5. Note that the value of the basic reproduction number implies a certain level of transmissibility (which is in this case representative of (seasonal) influenza). The outbreak probability was computed as the product of three quantities: (a) the observed proportion of participants within a certain age group, who owned or touched pets, livestock or/and poultry, (b) the probability that an infection does not die out, given that it starts in a particular age group, and (c) the size of the age group proportional to the total population. The outbreak probability was calculated assuming either age-heterogeneous or age-homogeneous social contact patterns.

## Results

Of 1768 survey respondents, 59.4% and 49.4%, respectively, owned and touched at least one animal (with 0.7% and 2.5% of missingness, respectively). Most of the reported animals were pets: 905 (51.2%) and 812 (45.9%) participants owned and touched pets, respectively (**Table** 1). While many participants had chickens at home (14.9%), only few touched poultry, and less than 5% owned or touched livestock (**Table** 1).

**Table 1:** Number and proportion of participants who reported owning and touching pets (cats, dogs, fish), livestock (horses, sheep, cows or pigs), poultry (chicken, turkeys, pigeons) and other animals* in Flanders, Belgium, 2010–2011(n = 1768).

	Owning (n = 1768)	Touching (n = 1768)
Pets	905 (51.19%)	812 (45.93%)
Cats	503 (28.45%)	450 (25.45%)
Dogs	437 (24.72%)	491 (27.77%)
Fish	267 (15.05%)	6 (0.34%)
Livestock	82 (4.64%)	45 (2.55%)
Horses	53 (3.00%)	32 (1.81%)
Sheep	21 (1.19%)	6 (0.34%)
Cows	18 (1.02%)	10 (0.56%)
Pigs	9 (0.51%)	1 (0.06%)
Poultry	272 (15.38%)	38 (2.15%)
Chickens	264 (14.93%)	29 (1.64%)
Pigeons	23 (1.30%)	10 (0.56%)
Turkeys	5 (0.28%)	0 (0.00%)

*Out of total participants, 326 (18.44%) and 117 (6.62%) participants owned/touched other animals.

### Modeling animal ownership

Animal ownership was significantly associated with age and household size, summarized in **Table 2**. The Hosmer-Lemeshow test [14] indicated that the model fitted the observed data well (p = 0.926). Participants aged 6–64 years were more likely to have animals in their household compared to participants aged 0–5 years (reference), whereas there was no significant difference between participants aged >64 years and reference. Participants living in a single person household were less likely to own animals than participants living in larger households.

**Table 2:** Multiple-logistic regression model for animal ownership in Flanders, Belgium, 2010–2011 (n = 1756).

Category	Sample size	Parameter estimates (SE^†^)	OR^†^	95% CI^†^ of OR	P value
**Age**					**<0.001**
0–5 years*	174		1.00		
6–11 years	127	0.88 (0.27)	2.41	[1.43, 4.06]	
12–17 years	79	0.72 (0.30)	2.05	[1.13, 3.72]	
18–44 years	621	0.59 (0.18)	1.81	[1.27, 2.60]	
45–64 years	468	0.67 (0.20)	1.95	[1.31, 2.88]	
65+ years	287	-0.31 (0.32)	0.73	[0.39, 1.36]	
**Household size**					**<0.001**
1*	98		1.00		
2	312	0.78 (0.24)	2.19	[1.38, 3.48]	
3	328	1.23 (0.24)	3.44	[2.14, 5.52]	
4	439	1.44 (0.24)	4.21	[2.62, 6.74]	
≥5	218	1.48 (0.27)	4.41	[2.60, 7.48]	
Missing	361	0.58 (0.31)	1.78	[0.97, 3.27]	

*Reference Category.

^†^OR = Odds Ratio, SE = Standard Error and CI = Confidence Interval.

Further results show that owners of one particular type of animals are more likely to own also other type of animals (see **Tables A-C in S2 File**). Further details on these results can be found in **S2 File**.

### Modeling animal touching

The final model for animal touching incorporated the main effects age and animal ownership, as well as their interaction, as summarized in **Table 3** (Hosmer-Lemeshow goodness-of-fit p = 0.999). Within the group of animal owners, participants of age 18–64 years were more likely to touch animals than children of age 0–5 years (reference), whereas there was no significant difference between the other age groups and the reference. As expected, the odds of touching animals was significantly higher for animal owners as compared to participants without animals in their household (“non-animal owners”).

**Table 3:** Multiple-logistic regression model for animal touching^†^ in Flanders, Belgium, 2010–2011 (n = 1722).

Covariate	Sample size	Parameter estimate (SE^‡^)	OR^‡^	95% CI^‡^ of OR	P value
**Age**					**<0.001**
0–5 years*	170		1.00		
6–11 years	125	-0.21 (0.30)	0.81	[0.45, 1.45]	
12–17 years	79	0.18 (0.35)	1.19	[0.60, 2.38]	
18–44 years	615	0.76 (0.25)	2.15	[1.32, 3.47]	
45–64 years	456	0.90 (0.26)	2.45	[1.47, 4.10]	
65+ years	277	0.03 (0.31)	1.03	[0.56, 1.88]	
**Animal ownership**					**<0.001**
Owner *	1037		1.00		
Not owner	685	-2.71 (0.43)	0.07	[0.03, 0.15]	
**Age: Animal Ownership**					**0.038**
6–11 years: Not owner	28	0.75 (0.69)	2.11	[0.55, 8.11]	
12–17 years: Not owner	20	0.79 (0.73)	2.20	[0.53, 9.21]	
18–44 years: Not owner	212	-0.39 (0.49)	0.68	[0.26, 1.76]	
45–64 years: Not owner	166	-0.94 (0.52)	0.39	[0.14, 1.09]	
65+ years: Not owner	188	-0.34 (0.55)	0.71	[0.24, 2.10]	

*Reference Category.

^†^We included animal ownership as a covariate.

^‡^OR = Odds Ratio, SE = Standard Error and CI = Confidence Interval.

Subsequently, we included cat, dog, livestock and poultry ownership separately as possible factors. The results of these analyses are shown (see **Tables A-D in S3 File**) and explained in **S3 File**.

### Modeling number of contacts

The mean number of social contacts for all participants was 13.5 with variance 116.8, a clear sign of overdispersion. Our final weighted-negative binomial model retained interaction effects of age and weekday, province and gender, animal ownership and weekday, and main effects only of household size and holiday period (**Table 4**). The overdispersion parameter was estimated at 2.43 (95% CI [2.24, 2.62]), indicating significant overdispersion.

**Table 4:** Weighted-negative binomial regression for the total number of contacts^†^ in Flanders, Belgium, 2010–2011 (n = 1742).

Covariate	Sample size	Median (IQR^§^)	Parameter estimates (SE^§^)	RNC^§^	95% CI^§^ for RNC	P-value
**Age**						**0.002**
0–5 years*	174	10.00 (11.00)		1.00		
6–11 years	127	18.00 (20.00)	0.56 (0.18)	1.76	[1.24, 2.51]	
12–17 years	79	15.00 (19.50)	0.47 (0.18)	1.61	[1.13, 2.30]	
18–44 years	621	11.00 (11.00)	0.58 (0.14)	1.79	[1.37, 2.33]	
45–64 years	466	11.00 (10.00)	0.59 (0.14)	1.80	[1.35, 2.37]	
65+ years	275	7.00 (9.50)	0.68 (0.18)	1.98	[1.38, 2.84]	
**Household size**						**<0.001**
1*	98	8.00 (10.00)		1.00		
2	312	10.00 (10.00)	0.15 (0.08)	1.17	[1.00, 1.35]	
3	328	11.00 (11.00)	0.21 (0.08)	1.24	[1.06, 1.44]	
4	439	13.00 (12.00)	0.29 (0.08)	1.34	[1.16, 1.56]	
≥5	218	14.00 (15.00)	0.39 (0.08)	1.48	[1.26, 1.74]	
Missing	347	7.00 (10.00)	0.03 (0.10)	1.04	[0.84, 1.27]	
**Animal ownership**						**0.002**
Owner*	1042	11.00 (12.00)		1.00		
Not owner	700	10.00 (11.00)	-0.24 (0.07)	0.79	[0.68, 0.91]	
**Gender**						**0.483**
Female*	930	11.00 (12.00)		1.00		
Male	812	11.00 (11.00)	0.05 (0.06)	1.05	[0.92, 1.19]	
**Province** ^‡^						**0.024**
Antwerp*	487	11.00 (11.00)		1.00		
Limburg	264	11.00 (13.25)	0.18 (0.07)	1.20	[1.04, 1.39]	
East Flanders	407	10.00 (11.00)	0.05 (0.06)	1.05	[0.93, 1.19]	
Flemish Brabant	257	10.00 (13.00)	0.20 (0.07)	1.22	[1.05, 1.41]	
West Flanders	327	11.00 (10.00)	0.14 (0.07)	1.15	[1.00, 1.32]	
**Weekday indicator**						**<0.001**
Weekend*	419	10.00 (10.00)		1.00		
Weekday	1323	11.00 (12.00)	0.62 (0.15)	1.85	[1.37, 2.48]	
**Holiday indicator**						**0.007**
Regular period*	1632	11.00 (12.00)		1.00		
Holiday period	110	8.00 (9.00)	-0.20 (0.07)	0.82	[0.71, 0.94]	
**Age: Weekday indicator**						**<0.001**
6–11 years: Weekday	96	20.00 (23.00)	-0.28 (0.21)	0.75	[0.50, 1.13]	
12–17 years: Weekday	58	18.50 (20.00)	-0.20 (0.21)	0.82	[0.54, 1.24]	
18–44 years: Weekday	466	12.00 (11.00)	-0.69 (0.16)	0.50	[0.37, 0.69]	
45–64 years: Weekday	358	11.00 (11.00)	-0.76 (0.16)	0.47	[0.34, 0.65]	
65+ years: Weekday	221	7.00 (9.00)	-1.13 (0.18)	0.32	[0.22, 0.46]	
**Province: Gender**						**0.020**
Limburg: Male	124	10.00 (10.75)	0.04 (0.11)	1.04	[0.84, 1.29]	
East Flanders: Male	176	11.00 (11.50)	-0.02 (0.10)	0.98	[0.81, 1.18]	
Flemish-Brabant: Male	121	14.00 (13.00)	-0.32 (0.11)	0.72	[0.58, 0.90]	
West Flanders: Male	171	15.00 (14.00)	-0.09 (0.10)	0.91	[0.75, 1.11]	
**Animal ownership: Weekday indicator**						**0.003**
Not an owner: Weekday	530	10.50 (11.00)	0.26 (0.08)	1.30	[1.09, 1.53]	
Dispersion = 2.43 (SE = 0.10), 95% CI [2.24, 2.62]						

*Reference Category.

^†^We included animal ownership as a covariate.

^‡^We excluded 14 observations with missing provinces because of not being enough for estimation.

^§^IQR = Inter-quartile range, RNC = Relative Number of Contact, SE = Standard Error and CI = Confidence Interval.

During the weekend, participants >5 years made significantly more contacts than children aged 0–5 years, while on a weekday participants >17 years made fewer contacts than children aged 0–5 years. Participants living with other people in the household had significantly more social contacts than participants living alone. Overall, participants had more social contacts on a weekday than on a weekend day, and during a regular period compared to a holiday period. Animal owners had more social contacts than non-animal owners during the weekend, but this difference disappeared on weekdays. Further details on these analyses can be found in **S4 File** (see **Table A in S4 File**).

A minority of 106 (6.2%) participants made no physical contacts on the assigned day. We found significant main effects on physical contacts of age, household size, animal ownership, province and weekday (see **Tables B and C in S4 File**).

#### Major outbreak probability of zoonoses with flu-like transmissibility, conditional on their presence in animal groups

**Fig 1:**
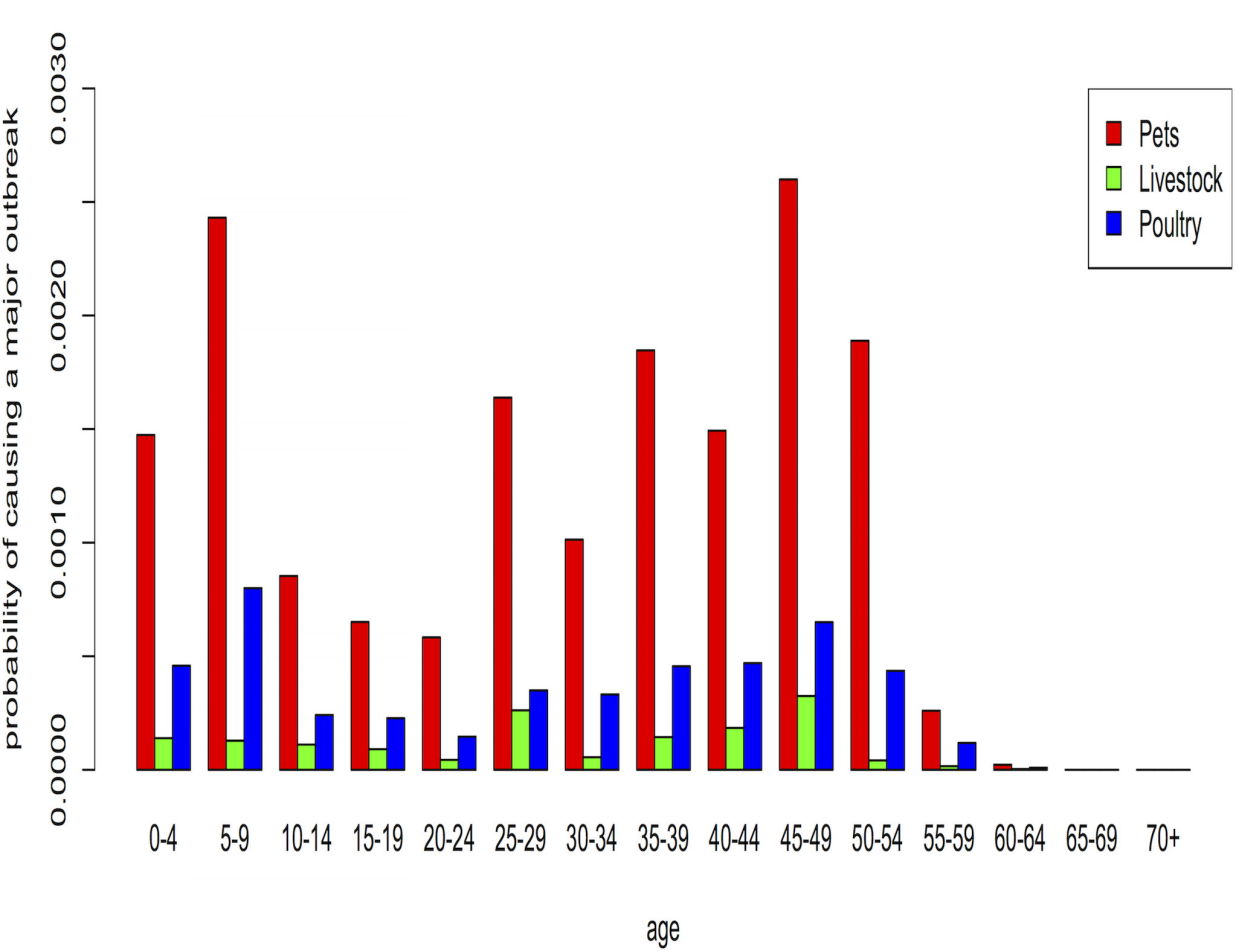
The probability of a human-transmissible pathogen causing a major outbreak in each age group, with owning of pets, livestock and poultry as exposure of pathogen spillover (assuming realistic—heterogeneously distributed—contacts by age).

**Fig 2:**
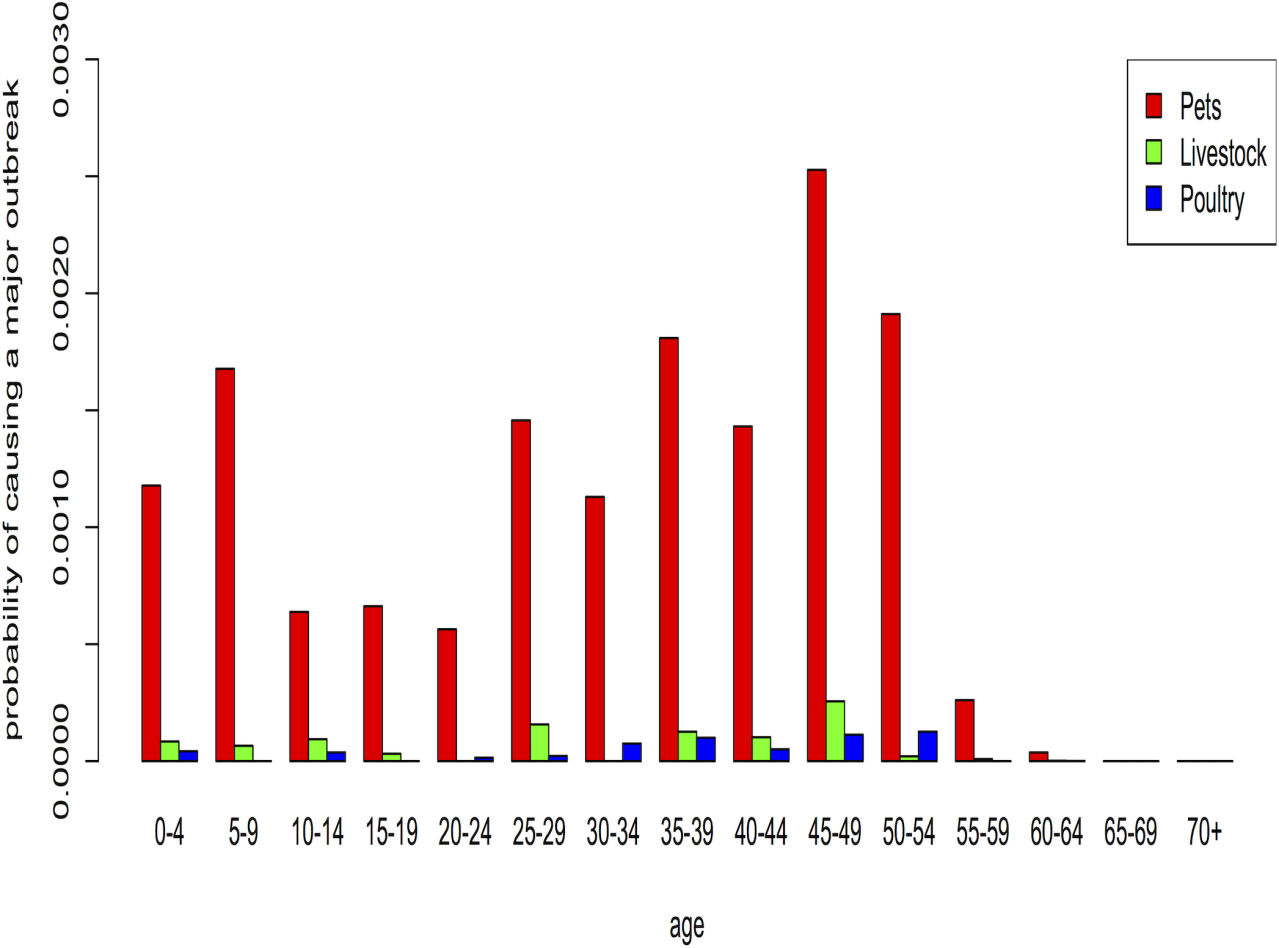
The probability of a human-transmissible pathogen causing a major outbreak in each age group, with touching of pets, livestock and poultry as exposure of pathogen spillover (assuming realistic—heterogeneously distributed—contacts by age).

First, we assumed age-heterogeneous social contact patterns to calculate the probability of a human-transmissible pathogen causing a major zoonotic outbreak (“probability of causing a major zoonotic outbreak”) in the different age groups. Pathogen spillover from animals to humans was assumed to occur only by owning and touching of pets, livestock and poultry. Given these circumstances and the assumption on pathogen transmissibility through our chosen basic reproduction number (i.e. influenza-like), **Figs** 1 and 2 show the probability that a major outbreak occurs through ownership and touching, respectively. In the context of Flanders the overall probability of a major outbreak is small, and pathogens originating from pets would pose a greater risk compared to poultry or livestock pathogens. If direct contact with live animals would be required, the relative risk posed by poultry or livestock pathogens is negligible, simply because far fewer people have direct contacts with such animals than with pets in Belgium. If ownership would be the main driver, then poultry owners are more likely to be instrumental in causing a major outbreak than livestock owners. In general, older adults (>55 years) have the lowest probability to cause a major zoonotic outbreak, whereas younger adults (25–54 years) and children (0–9 years) are most likely to cause a zoonotic outbreak.

To study the impact of age-heterogeneous social contact patterns, we also performed the analysis with homogeneous mixing patterns. In general, we observed similar results with both mixing assumptions. Nonetheless the probability for children (0–4 years) and adults > 55 years to cause a major outbreak increased with homogeneous mixing as compared to heterogeneous mixing. The epidemic potential of children, teenagers and young adults (5–24 years) decreased under these conditions (see **S2 and S3 Figs**).

## Discussion

Many pathogens in human populations have animal reservoirs [2], hence it is important to understand human-animal interactions in the context of disease transmission and social contact patterns.

Empirical studies have shown the potential benefits of animal ownership on human health [17–21]. However, pets can carry bacteria, viruses, parasites and fungi causing illness in humans. People may acquire animal-borne diseases when they are bitten, scratched or touched by animals. Therefore, we presented the results of a large survey documenting social contact patterns together with data on owning and touching animals.

In a HIC with little private agricultural activity like Belgium, it is expected that people generally interact more with pets (dog, cat, fish) than with livestock or poultry. The most popular pets were dogs and cats. Ownership was slightly more (28.5% compared to 24.7%), and touching slightly less frequent (25.5% compared to 27.8%) for cats compared to dogs. Household size was a common factor for owning animals, pets, cats or dogs. Moreover, age was also a common factor for owning animals, pets or cats but not for owning dogs. Participants aged 6–64 years were more likely to own animals than children aged 0–5 years, as were participants of larger households compared to those living alone. The most common reason for pet ownership is companionship [22]. Although elderly people generally have fewer social contacts and would benefit the most from pet companionship to boost their optimism and exercise [21], they were less likely to own animals (especially cats) according to our study, even if they were living alone. This could be because pet care might be a burden for elderly. Furthermore, they may outlive their pets and not replace them, or not take them with them when they move into a nursing home. Previous work suggested that there were gender differences in pet ownership [23]. In households of size two and above four, we also found significant effects of gender on pet ownership. According to our study, livestock and poultry owners were more likely to own pets (mostly cats and dogs). Note that British horse owners were previously shown more likely to own dogs [23].

Our study suggests that the probability of touching an animal during a randomly assigned day depended on the main and interaction effects of age and animal ownership. People who did not own animals had a low chance to touch an animal on a random day. Some but not all of the underlying factors are similar for pet, cat and dog touching. Touching of cats and dogs was not only affected by cat and dog ownership, respectively, but also by livestock and poultry ownership, respectively.

The results in this paper are based on the second large prospective social contact survey conducted in Belgium. Inter-human contact patterns relevant to infectious disease transmission have been analyzed in many earlier studies [7, 9, 10, 24,25]. The effects of age, household size, weekend and holidays we identified for the total number of contacts (see **Table 4**) are in line with earlier findings from the first Belgian survey [10, 16], but we also found an effect of animal ownership, gender and province. While Hens et al. (2009) [10] found no significant difference between males and females; we found significant gender differences that varied through the different provinces of Flanders. For both all and physical contacts, we found similar significant factors. Generally, animal owners have more social contacts than non-animal owners. In addition, we found dog ownership and poultry ownership to be associated with the total number of contacts but not with physical contacts. For middle-aged adults (45–64 years), owning a dog could be a good way of facilitating more social contacts on the weekend (see also [26–27].

Mathematical models of zoonotic outbreaks are of increasing interest, but many important gaps still remain. Previous research was done with a simple stochastic model of directly transmitted zoonoses [28]. Our study demonstrates a method to characterize the relative probability of a major zoonotic outbreak from different animals, by relating human-animal interactions with social contact frequencies between humans in different age groups. Key assumptions here were that (1) the probability is conditional on the pathogen’s presence in the animal group, and (2) pathogen spillover from animals to humans was driven by animal ownership and touching. We divided the study population by age and calculated the probability that a major outbreak would occur through animal contacts by age group. As a result, we found that children (0–9 years) and adults (25–54 years) were generally more likely to cause a major zoonotic outbreak.

A limitation of our study is that we focused on ownership and direct touching of animals as potential risk factors for infectious disease transmission. We did not attempt to survey potential exposure to animal excrements (e.g. due to gardening or vicinity of forests) or raw meat consumption. Nevertheless, our study is the first that analyses human-animal interactions in the context of inter-human contacts. Although the results apply to a high-income country setting, the survey and analytical methods can also be applied to low- and middle-income countries. The amount of missing data was limited and therefore complete case analysis was used.

Many published studies have used paper contact diaries, as we did here, to quantify potentially infectious contacts between people [7–12, 16, 29]. Contact diaries have two primary limitations [29]. First, it is difficult to assess potential biases in recollection and reporting, especially for participants with many contacts in different places. The second limitation is that direct conversation or physical contacts potentially limit the reported encounters to a subset of all the social encounters that could enable transmission. Despite these limitations, many authors have suggested that contact diaries have important pragmatic advantages to quantify social contacts relevant to infectious disease transmission in large population settings [29]. Finally, our results need to be interpreted in the context of a densely populated HIC in Europe. Similar studies in both similar and different contexts are needed to confirm these findings and test our methods.

## Supporting Information

S1 Fig The sampled questions added to the contact diaries to gather information about human-animal interactions. (TIFF)

S2 Fig The probability of a human-transmissible pathogen causing a major outbreak with flu-like transmissibility and with owning of pets, livestock and poultry as exposure of pathogen spillover by age (assuming homogeneously distributed contacts by age). (TIFF)

S3 Fig The probability of a human-transmissible pathogen causing a major outbreak with flu-like transmissibility and with touching of pets, livestock and poultry as exposure of pathogen spillover by age (assuming homogeneously distributed contacts by age). (TIFF)

S1 File Demographic factors. (DOCX)

S2 File Modeling animal ownership. (DOCX)

S3 File Modeling animal touching. (DOCX)

S4 File Modeling number of contacts. (DOCX)

S5 File Major outbreak probability of zoonoses with flu-like transmissibility, conditional on their presence in animal groups. (DOCX)

S6 File Datasets. (ZIP)
