# Correction: Animal Ownership and Touching Enrich the Context of Social Contacts Relevant to the Spread of Human Infectious Diseases

**DOI:** 10.1371/journal.pone.0148718

**Published:** 2016-02-03

**Authors:** Yimer Wasihun Kifle, Nele Goeyvaerts, Kim Van Kerckhove, Lander Willem, Adam Kucharski, Christel Faes, Herwig Leirs, Niel Hens, Philippe Beutels

Dr. Adam Kucharski is not included in the author byline. He should be listed as the fifth author and affiliated with the Centre for the Mathematical Modelling of Infectious Diseases, London School of Hygiene & Tropical Medicine, London, UK. The contributions of this author are as follows: Analyzed the data, wrote the paper.

The correct citation is: Kifle YW, Goeyvaerts N, Van Kerckhove K, Willem L, Kucharski A, Faes C, et al. (2015) Animal Ownership and Touching Enrich the Context of Social Contacts Relevant to the Spread of Human Infectious Diseases. PLoS ONE 10(7): e0133461. doi:10.1371/journal.pone.0133461
