# Supplementary figures and images for: Animal Ownership and Touching Enrich the Context of Social Contacts Relevant to the Spread of Human Infectious Diseases
Source: PLoS One. 2015 Jul 20;10(7):e0133461. doi: 10.1371/journal.pone.0133461 (PMC4508096; doi:10.1371/journal.pone.0133461)

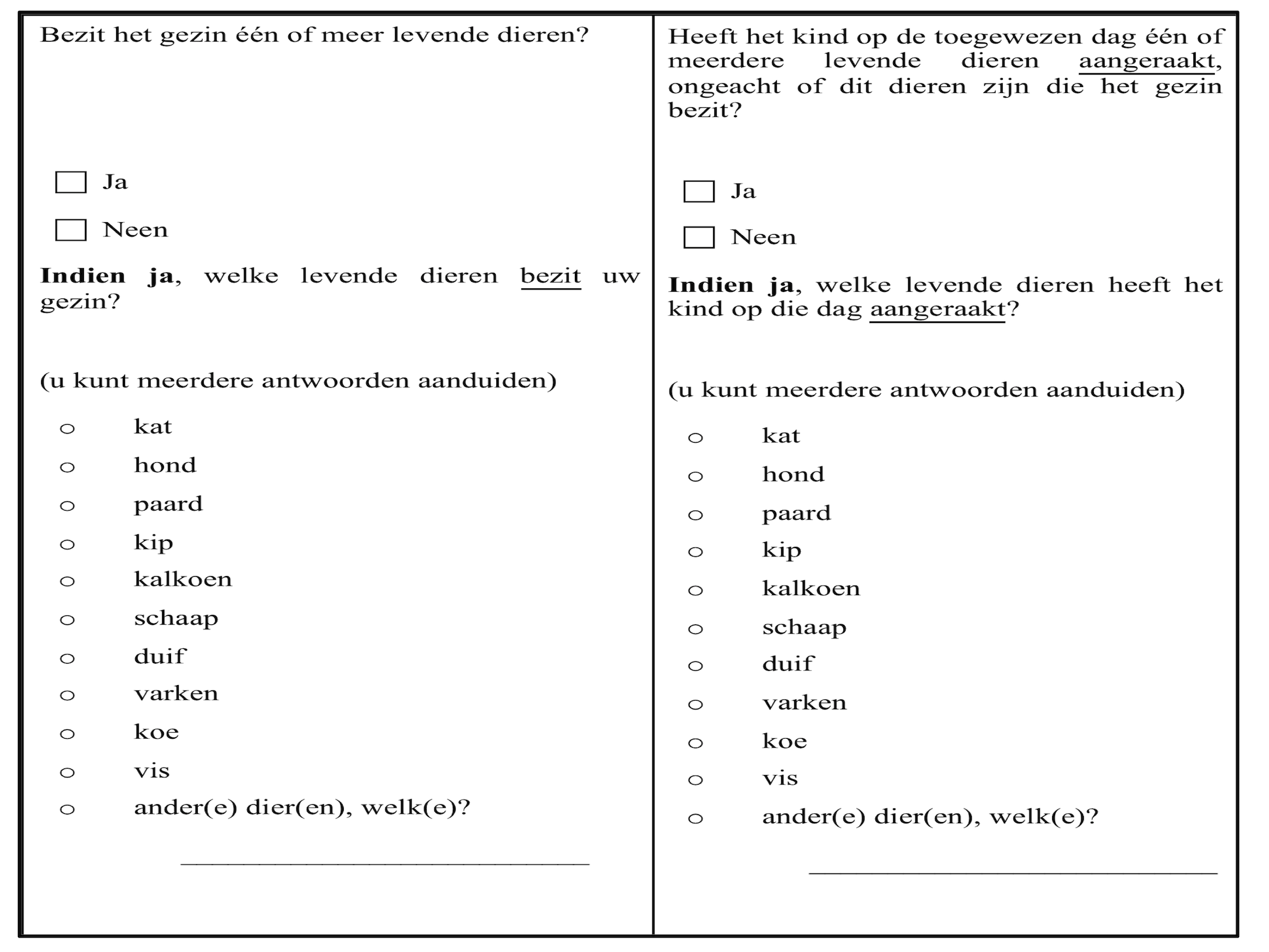

Supplement: S1 Fig — (TIFF) [file pone.0133461.s001.tiff]

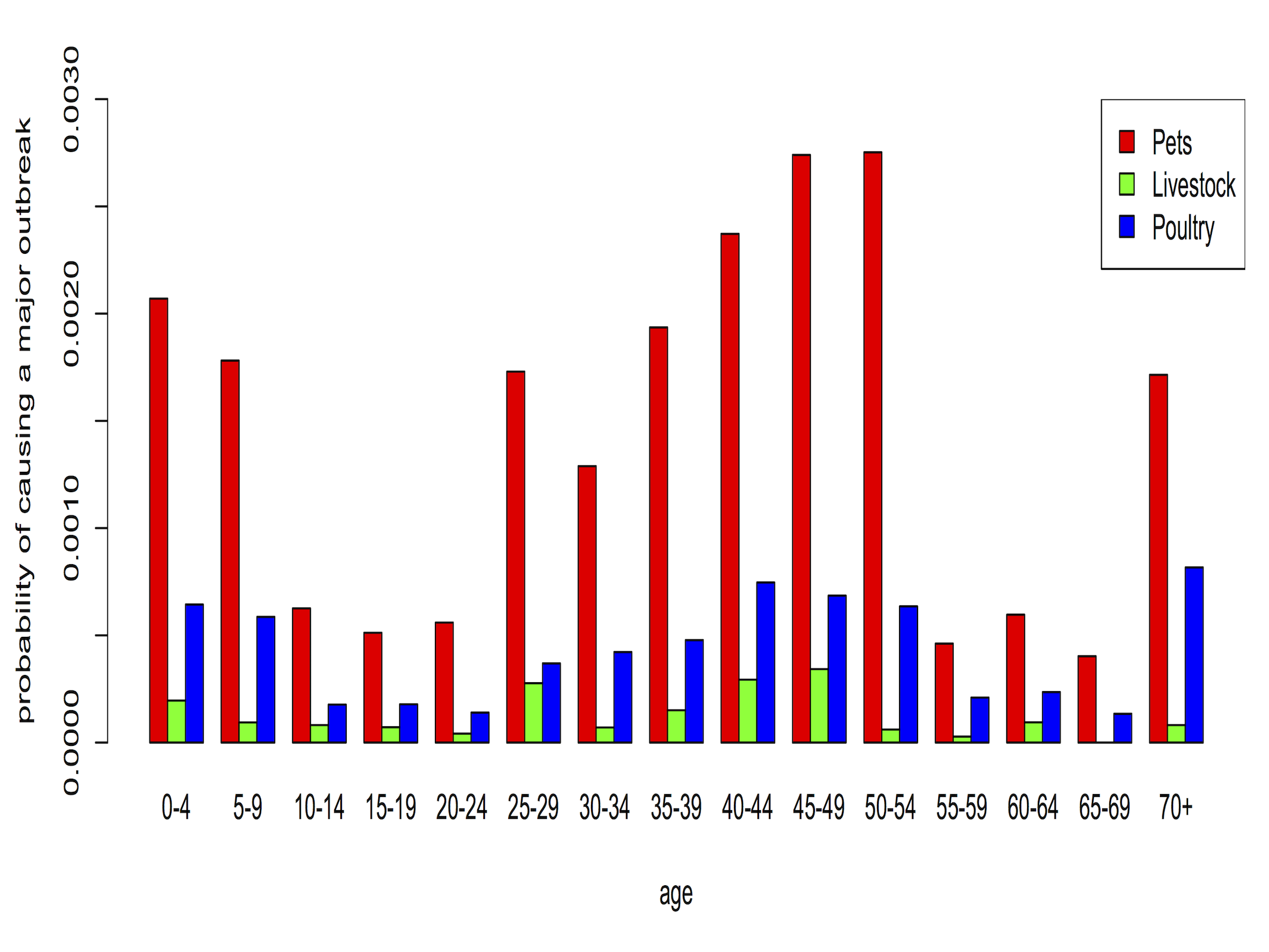

Supplement: S2 Fig — (TIFF) [file pone.0133461.s002.tiff]

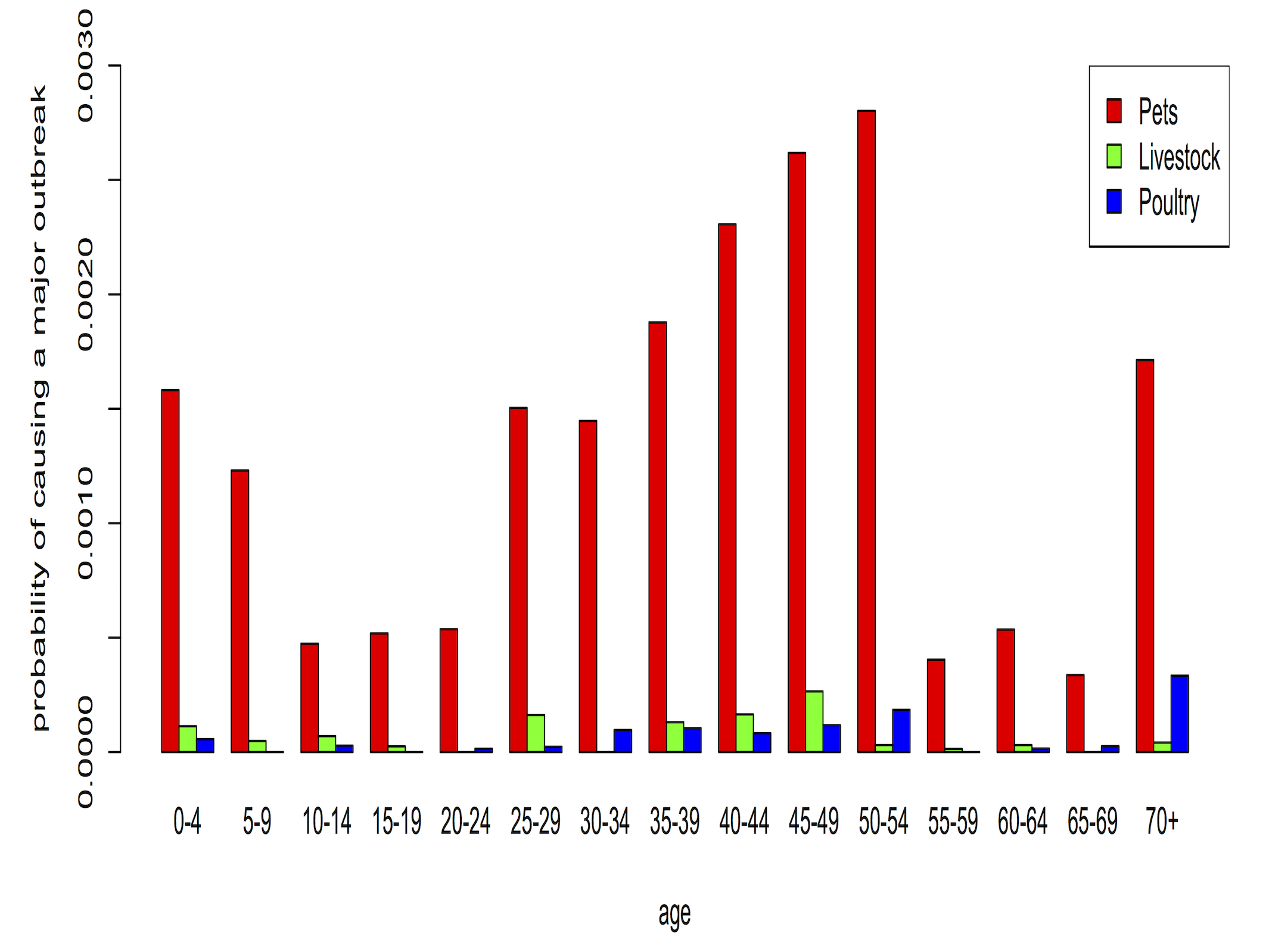

Supplement: S3 Fig — (TIFF) [file pone.0133461.s003.tiff]
