# Supplementary material for: Animal Ownership and Touching Enrich the Context of Social Contacts Relevant to the Spread of Human Infectious Diseases
Source: PLoS One. 2015 Jul 20;10(7):e0133461. doi: 10.1371/journal.pone.0133461 (PMC4508096; doi:10.1371/journal.pone.0133461)
Supplement: S1 File — (DOCX) [file pone.0133461.s004.docx]

## S1 File. Demographic factors

Educational attainment (maximum level of education completed) was categorized as no education, primary school, secondary education and higher education. Since the educational attainment was confounded by age in the survey, three separate variables were considered: educational attainment of children (0-12 years) (i.e. educational attainment of their mother), educational attainment of participants (≥13 years) who were studying during the survey, and educational attainment of participants (≥13 years) who were not studying during the survey. The Flemish region consists of five provinces (from west to east): West Flanders, East Flanders, Flemish Brabant, Antwerp and Limburg. Household sizes larger than four were categorized into a single group.
