# Supplementary material for: Animal Ownership and Touching Enrich the Context of Social Contacts Relevant to the Spread of Human Infectious Diseases
Source: PLoS One. 2015 Jul 20;10(7):e0133461. doi: 10.1371/journal.pone.0133461 (PMC4508096; doi:10.1371/journal.pone.0133461)
Supplement: S2 File — (DOCX) [file pone.0133461.s005.docx]

## S2 File. Modeling animal ownership

Further, pet ownership was associated with age, gender, household size, province, livestock ownership and poultry ownership, together with the interaction effect between household size and gender (**Table A**). Cat ownership was associated with age, household size, province, livestock ownership and poultry ownership (**Table B**). Finally, dog ownership was associated with household size, not being a student (≥13 years), livestock ownership and poultry ownership (**Table C**).

**Table A**: Multiple-logistic regression model for pet ownership in Flanders, Belgium, 2010-2011 (n=1756)

| Covariate | Sample size | Parameter estimate (SE^†^) | OR^†^ | 95% CI^†^ of OR | P value |
| --- | --- | --- | --- | --- | --- |
| **Age** |  |  |  |  | **0.008** |
| 0-5 years^*^ | 174 |  |  |  |  |
| 6-11 years | 127 | 0.51 (0.25) | 1.67 | [1.03, 2.70] |  |
| 12-17 years | 79 | 0.50 (0.29) | 1.65 | [0.93, 2.93] |  |
| 18-44 years | 621 | 0.48 (0.19) | 1.62 | [1.13, 2.33] |  |
| 45-64 years | 468 | 0.57 (0.20) | 1.78 | [1.19, 2.64] |  |
| 65+ years | 287 | -0.14 (0.33) | 0.87 | [0.45, 1.66] |  |
| **Gender** |  |  |  |  | **0.039** |
| Female^*^ | 942 |  |  |  |  |
| Male | 814 | -0.92 (0.46) | 0.40 | [0.16, 0.97] |  |
| **Household size** |  |  |  |  | **<0.001** |
| 1^*^ | 98 |  |  |  |  |
| 2 | 312 | 0.27 (0.32) | 1.31 | [0.70, 2.43] |  |
| 3 | 328 | 0.64 (0.32) | 1.91 | [1.01, 3.59] |  |
| 4 | 439 | 0.91 (0.32) | 2.49 | [1.34, 4.64] |  |
| ≥5 | 218 | 0.50 (0.36) | 1.65 | [0.82, 3.33] |  |
| Missing | 361 | -0.47 (0.39) | 0.62 | [0.29, 1.34] |  |
| **Province** |  |  |  |  | **0.022** |
| Antwerp^*^ | 487 |  |  |  |  |
| Limburg | 264 | -0.08 (0.17) | 0.92 | [0.67, 1.28] |  |
| East Flanders | 407 | 0.26 (0.14) | 1.30 | [0.98, 1.73] |  |
| Flemish Brabant | 257 | 0.11 (0.17) | 1.11 | [0.81, 1.54] |  |
| West Flanders | 327 | 0.34 (0.15) | 1.40 | [1.03, 1.89] |  |
| Missing | 14 | 1.33 (0.58) | 3.79 | [1.22, 11.71] |  |
| **Livestock ownership** |  |  |  |  | **<0.001** |
| Owner^*^ | 82 |  |  |  |  |
| Not owner | 1674 | -1.42 (0.34) | 0.24 | [0.12, 0.47] |  |
| **Poultry ownership** |  |  |  |  | **<0.001** |
| Owner^*^ | 272 |  |  |  |  |
| Not owner | 1484 | -0.90 (0.16) | 0.41 | [0.30, 0.55] |  |
| **Household size: Gender** |  |  |  |  | **0.037** |
| 2: Male | 126 | 1.01 (0.51) | 2.76 | [1.01, 7.54] |  |
| 3: Male | 158 | 0.92 (0.51) | 2.51 | [0.92, 6.85] |  |
| 4: Male | 199 | 0.56 (0.50) | 1.74 | [0.65, 4.64] |  |
| ≥5: male | 116 | 1.28 (0.54) | 3.60 | [1.24, 10.44] |  |
| Missing: male | 171 | 1.33 (0.52) | 3.77 | [1.37, 10.41] |  |

^*^Reference Category.

^†^OR=Odds Ratio, SE= Standard Error and CI= Confidence Interval.

**Table B**: Multiple-logistic regression model for cat ownership in Flanders, Belgium, 2010-2011 (n=1756)

| Covariate | Sample size | Parameter estimate (SE^†^) | | OR^†^ | 95% CI^†^ of OR | P value |
| --- | --- | --- | --- | --- | --- | --- |
| **Age** |  |  | |  |  | **0.013** |
| 0-5 years^*^ | 174 |  | | 1.00 |  |  |
| 6-11 years | 127 | 0.15 (0.26) | | 1.16 | [0.70, 1.91] |  |
| 12-17 years | 79 | 0.28 (0.29) | | 1.32 | [0.74, 2.34] |  |
| 18-44 years | 621 | 0.23 (0.19) | | 1.25 | [0.86, 1.84] |  |
| 45-64 years | 468 | 0.17 (0.21) | | 1.19 | [0.78, 1.81] |  |
| 65+ years | 287 | -0.98 (0.38) | | 0.37 | [0.18, 0.79] |  |
| **Household size** |  |  | |  |  | **0.011** |
| 1^*^ | 98 | |  |  |  |  |
| 2 | 312 | -0.01 (0.28) | | 0.99 | [0.57, 1.70] |  |
| 3 | 328 | 0.42 (0.27) | | 1.53 | [0.89, 2.61] |  |
| 4 | 439 | 0.59 (0.27) | | 1.80 | [1.06, 3.05] |  |
| ≥5 | 218 | 0.59 (0.29) | | 1.81 | [1.02, 3.21] |  |
| Missing | 361 | 0.14 (0.36) | | 1.15 | [0.57, 2.33] |  |
| **Province** |  |  | |  |  | **<0.001** |
| Antwerp^*^ | 487 |  | |  |  |  |
| Limburg | 264 | -0.19 (0.19) | | 0.83 | [0.57, 1.21] |  |
| East Flanders | 407 | 0.67 (0.15) | | 1.96 | [1.45, 2.65] |  |
| Flemish-Brabant | 257 | 0.20 (0.18) | | 1.22 | [0.86, 1.74] |  |
| West Flanders | 327 | 0.44 (0.17) | | 1.56 | [1.13, 2.15] |  |
| Missing | 14 | 1.70 (0.61) | | 5.47 | [1.67, 17.91] |  |
| **Livestock ownership** |  |  | |  |  | **0.003** |
| Owner^*^ | 82 |  | |  |  |  |
| Not owner | 1674 | -0.72 (0.24) | | 0.48 | [0.30, 0.78] |  |
| **Poultry ownership** |  |  | |  |  | **<0.001** |
| Owner^*^ | 272 |  | |  |  |  |
| Not owner | 1484 | -0.56 (0.15) | | 0.57 | [0.43, 0.76] |  |

^*^Reference Category.

^†^OR=Odds Ratio, SE= Standard Error and CI= Confidence Interval.

**Table C**: Multiple-logistic regression model for dog ownership in Flanders, Belgium, 2010-2011 (n=1756)

| Covariate | Sample size | Parameter estimate (SE^†^) | OR^†^ | 95% CI^†^ of OR | P value |
| --- | --- | --- | --- | --- | --- |
| **Household size** |  |  |  |  | **<0.001** |
| 1^*^ | 98 |  | 1.00 |  |  |
| 2 | 312 | 1.08 (0.33) | 2.94 | [1.55, 5.60] |  |
| 3 | 328 | 0.94 (0.33) | 2.56 | [1.33, 4.90] |  |
| 4 | 439 | 0.92 (0.33) | 2.51 | [1.32, 4.79] |  |
| ≥5 | 218 | 1.06 (0.35) | 2.88 | [1.45, 5.74] |  |
| Missing | 361 | -0.01 (0.34) | 1.00 | [0.51, 1.95] |  |
| **Participants of age ≥13 years and not student indicator** |  |  |  |  | **0.03** |
| No^*^ | 446 |  | 1.00 |  |  |
| Yes | 1310 | 0.32 (0.15) | 1.38 | [1.03, 1.84] |  |
| **Livestock ownership** |  |  |  |  | **<0.001** |
| Owner^*^ | 82 |  | 1.00 |  |  |
| Not owner | 1674 | -1.93 (0.26) | 0.14 | [0.09, 0.24] |  |
| **Poultry ownership** |  |  |  |  | **0.014** |
| Owner^*^ | 272 |  | 1.00 |  |  |
| Not owner | 1484 | -0.38 (0.15) | 0.68 | [0.50, 0.92] |  |

^*^Reference Category.

^†^OR=Odds Ratio, SE= Standard Error and CI= Confidence Interval.
