# Supplementary material for: Animal Ownership and Touching Enrich the Context of Social Contacts Relevant to the Spread of Human Infectious Diseases
Source: PLoS One. 2015 Jul 20;10(7):e0133461. doi: 10.1371/journal.pone.0133461 (PMC4508096; doi:10.1371/journal.pone.0133461)
Supplement: S3 File — (DOCX) [file pone.0133461.s006.docx]

## S3 File. Modeling animal touching

We found that animal touching was significantly associated with the total number of contacts, age, dog ownership and livestock ownership. This was also associated with the two-way interactions between the total number of contacts and cat ownership, age and poultry ownership, cat and dog ownership, and cat and livestock ownership (**Table** **A**). Moreover, pet touching was associated with the main effects of household size, being a student (≥13 years), not being a student (≥13 years), dog ownership, livestock ownership and weekday. There were also associations with the two-way interactions of cat and livestock ownership, and cat and dog ownership (**Table** **B**). Cat touching was associated with age, cat ownership, livestock ownership and the interaction between age and cat ownership (**Table** **C**). Dog touching was associated with age, household size, dog ownership, poultry ownership, and holiday period (**Table** **D**).

**Table** **A**: Multiple-logistic regression model for animal touching^†^ in Flanders, Belgium, 2010-2011 (n=1722)

| Covariate | Sample size | Parameter estimate (SE^‡^) | OR^‡^ | 95% CI^‡^ of OR | P value |
| --- | --- | --- | --- | --- | --- |
| **TNC**^‡^ |  | -0.03 (0.01) | 0.98 | [0.96, 0.99] | **0.016** |
| **Age** |  |  |  |  | **0.004** |
| 0-5 years^*^ | 170 |  | 1.00 |  |  |
| 6-11 years | 125 | 0.77 (0.77) | 2.15 | [0.47, 9.77] |  |
| 12-17 years | 79 | 0.75 (0.83) | 2.12 | [0.42, 10.78] |  |
| 18-44 years | 615 | 1.62 (0.60) | 5.07 | [1.55, 16.59] |  |
| 45-64 years | 456 | 2.20 (0.63) | 9.02 | [2.63, 30.96] |  |
| 65+ years | 277 | 2.29 (0.72) | 9.88 | [2.42, 40.38] |  |
| **Cat ownership** |  |  |  |  | **0.311** |
| Owner^*^ | 499 |  | 1.00 |  |  |
| Not owner | 1223 | 0.75 (0.73) | 2.12 | [0.50, 8.94] |  |
| **Dog ownership** |  |  |  |  | **<0.001** |
| Owner^*^ | 433 |  | 1.00 |  |  |
| Not owner | 1289 | -1.70 (0.53) | 0.18 | [0.07, 0.51] |  |
| **Livestock ownership** |  |  |  |  | **0.006** |
| Owner^*^ | 82 |  | 1.00 |  |  |
| Not owner | 1640 | 1.59 (0.56) | 4.92 | [1.66, 14.63] |  |
| **Poultry ownership** |  |  |  |  | **0.21** |
| Owner^*^ | 266 |  | 1.00 |  |  |
| Not owner | 1456 | 0.74 (0.58) | 2.09 | [0.67, 6.48] |  |
| **TNC: Cat ownership** |  |  |  |  | **0.019** |
| TNC: Not owner |  | 0.03 (0.01) | 1.03 | [1.01, 1.06] |  |
| **Age: Poultry ownership** |  |  |  |  | **0.007** |
| 6-11 years: Not owner | 102 | -0.48 (0.86) | 0.62 | [0.12, 3.32] |  |
| 12-17 years: Not owner | 60 | 0.21 (0.93) | 1.23 | [0.20, 7.66] |  |
| 18-44 years: Not owner | 520 | -0.96 (0.66) | 0.38 | [0.10, 1.41] |  |
| 45-64 years: Not owner | 382 | -1.63 (0.69) | 0.2 | [0.05, 0.75] |  |
| 65+ years: Not owner | 248 | -2.46 (0.78) | 0.09 | [0.02, 0.39] |  |
| **Cat ownership: Dog ownership** |  |  |  |  | **0.001** |
| Not owner (cat): Not owner (dog) | 922 | -2.10 (0.57) | 0.12 | [0.04, 0.38] |  |
| **Cat ownership: Livestock ownership** |  |  |  |  | **<0.001** |
| Not owner (cat): Not owner (livestock) | 922 | -2.51 (0.75) | 0.08 | [0.02, 0.35] |  |

^*^Reference Category.

^†^We included cat, dog, livestock and poultry ownership as a covariate instead of animal ownership.

^‡^TNC=Total Number of Contacts, OR=Odds Ratio, SE=Standard Error and CI=Confidence Interval.

**Table** **B**: Multiple-logistic regression model for pet touching in Flanders, Belgium, 2010-2011 (n=1722)

| Covariate | Sample size | Parameter estimate (SE^†^) | OR^†^ | 95% CI^†^ of OR | P value |
| --- | --- | --- | --- | --- | --- |
| **Household size** |  |  |  |  | **0.029** |
| 1^*^ | 98 |  | 1.00 |  |  |
| 2 | 312 | 0.08 (0.35) | 1.08 | [0.54, 2.16] |  |
| 3 | 328 | -0.09 (0.36) | 0.91 | [0.45, 1.85] |  |
| 4 | 439 | -0.12 (0.36) | 0.88 | [0.44, 1.78] |  |
| ≥5 | 218 | -0.29 (0.40) | 0.75 | [0.34, 1.63] |  |
| Missing | 361 | -0.74 (0.36) | 0.48 | [0.24, 0.96] |  |
| **Participants of age ≥13 years and student indicator** |  |  |  |  | **0.002** |
| No^*^ | 1591 |  | 1.00 |  |  |
| Yes | 131 | 1.03 (0.34) | 2.80 | [1.45, 5.41] |  |
| **Participants of age ≥13 years and not student indicator** |  |  |  |  | **<0.001** |
| No^*^ | 440 |  | 1.00 |  |  |
| Yes | 1282 | 1.05 (0.23) | 2.87 | [1.83, 4.50] |  |
| **Cat ownership** |  |  |  |  | **0.742** |
| Owner^*^ | 499 |  | 1.00 |  |  |
| Not owner | 1223 | 0.24 (0.71) | 1.27 | [0.31, 5.12] |  |
| **Dog ownership** |  |  |  |  | **<0.001** |
| Owner^*^ | 433 |  | 1.00 |  |  |
| Not owner | 1289 | -1.90 (0.51) | 0.15 | [0.05, 0.41] |  |
| **Livestock ownership** |  |  |  |  | **0.001** |
| Owner^*^ | 82 |  | 1.00 |  |  |
| Not owner | 1640 | 1.70 (0.52) | 5.45 | [1.98, 15.03] |  |
| **Weekday indicator** |  |  |  |  | **0.031** |
| Weekend^*^ | 416 |  | 1.00 |  |  |
| Weekday | 1306 | -0.39 (0.18) | 0.68 | [0.48, 0.96] |  |
| **Cat ownership: Livestock ownership** |  |  |  |  | **0.034** |
| Not owner (cat): Not owner (livestock) | 1184 | -1.61 (0.76) | 0.20 | [0.04, 0.90] |  |
| **Cat ownership: Dog ownership** |  |  |  |  | **<0.001** |
| Not owner (cat): Not owner (dog) | 922 | -2.52 (0.56) | 0.08 | [0.03, 0.24] |  |

^*^Reference Category.

^†^OR=Odds Ratio, SE= Standard Error and CI= Confidence Interval.

**Table** **C**: Multiple-logistic regression model for cat touching in Flanders, Belgium, 2010-2011 (n=1722)

| Covariate | Sample size | Parameter estimate (SE^†^) | OR^†^ | 95% CI^†^ of OR | P value |
| --- | --- | --- | --- | --- | --- |
| **Age** |  |  |  |  | **0.002** |
| 0-5 years^*^ | 170 |  | 1.00 |  |  |
| 6-11 years | 125 | 0.20 (0.47) | 1.23 | [0.49, 3.06] |  |
| 12-17 years | 79 | -0.16 (0.49) | 0.85 | [0.33, 2.22] |  |
| 18-44 years | 615 | 1.10 (0.37) | 2.99 | [1.46, 6.13] |  |
| 45-64 years | 456 | 1.10 (0.40) | 3.01 | [1.38, 6.57] |  |
| 65+ years | 277 | 0.26 (0.52) | 1.30 | [0.47, 3.58] |  |
| **Cat ownership** |  |  |  |  | **<0.001** |
| Owner^*^ | 499 |  | 1.00 |  |  |
| Not owner | 1223 | -4.24 (0.59) | 0.01 | [0.00, 0.05] |  |
| **Livestock ownership** |  |  |  |  | **<0.001** |
| Owner^*^ | 82 |  | 1.00 |  |  |
| Not owner | 1640 | 1.40 (0.34) | 4.07 | [2.09, 7.92] |  |
| **Age: Cat ownership** |  |  |  |  | **0.030** |
| 6-11 years: Not owner | 82 | -0.11 (0.91) | 0.90 | [0.15, 5.30] |  |
| 12-17 years: Not owner | 48 | 1.19 (0.88) | 3.27 | [0.58, 18.34] |  |
| 18-44 years: Not owner | 409 | -1.00 (0.68) | 0.37 | [0.10, 1.40] |  |
| 45-64 years: Not owner | 320 | -1.27 (0.73) | 0.28 | [0.07, 1.17] |  |
| 65+ years: Not owner | 246 | -0.09 (0.79) | 0.92 | [0.19, 4.33] |  |

^*^Reference Category.

^†^OR=Odds Ratio, SE= Standard Error and CI= Confidence Interval.

**Table** **D**: Multiple-logistic regression model for dog touching in Flanders, Belgium, 2010-2011 (n=1722)

| Covariate | Sample size | Parameter estimate (SE^†^) | OR^†^ | 95% CI^†^ of OR | P value |
| --- | --- | --- | --- | --- | --- |
| **Age** |  |  |  |  | **0.032** |
| 0-5 years^*^ | 170 |  | 1.00 |  |  |
| 6-11 years | 125 | 0.78 (0.46) | 2.17 | [0.88, 5.37] |  |
| 12-17 years | 79 | 1.50 (0.51) | 4.47 | [1.66, 12.04] |  |
| 18-44 years | 615 | 1.01 (0.35) | 2.76 | [1.38, 5.51] |  |
| 45-64 years | 456 | 1.07 (0.38) | 2.92 | [1.39, 6.10] |  |
| 65+ years | 277 | 0.77 (0.63) | 2.16 | [0.63, 7.49] |  |
| **Household size** |  |  |  |  | **0.014** |
| 1^*^ | 95 |  | 1.00 |  |  |
| 2 | 308 | 0.36 (0.39) | 1.43 | [0.67, 3.06] |  |
| 3 | 320 | 0.001 (0.40) | 1.00 | [0.45, 2.20] |  |
| 4 | 436 | -0.24 (0.40) | 0.79 | [0.36, 1.73] |  |
| 5 and above | 216 | -0.76 (0.46) | 0.47 | [0.19, 1.15] |  |
| Missing | 347 | -0.80 (0.58) | 0.45 | [0.14, 1.42] |  |
| **Dog ownership** |  |  |  |  | **<0.001** |
| Owner^*^ | 433 |  | 1.00 |  |  |
| Not owner | 1289 | -4.89 (0.22) | 0.01 | [0.00, 0.01] |  |
| **Poultry ownership** |  |  |  |  | **0.031** |
| Owner^*^ | 266 |  | 1.00 |  |  |
| Not owner | 1456 | 0.55 (0.26) | 1.73 | [1.04, 2.89] |  |
| **Holiday indicator** |  |  |  |  | **0.036** |
| Regular period^*^ | 1614 |  | 1.00 |  |  |
| Holiday period | 108 | -0.79 (0.38) | 0.45 | [0.21, 0.96] |  |

^†^OR=Odds Ratio, SE= Standard Error and CI= Confidence Interval.

^*^Reference Category.
