# Supplementary material for: Animal Ownership and Touching Enrich the Context of Social Contacts Relevant to the Spread of Human Infectious Diseases
Source: PLoS One. 2015 Jul 20;10(7):e0133461. doi: 10.1371/journal.pone.0133461 (PMC4508096; doi:10.1371/journal.pone.0133461)
Supplement: S4 File — (DOCX) [file pone.0133461.s007.docx]

## S4 File. Modeling number of contacts

The number of contacts was associated with age, household size, province, weekday and holiday period, together with two-way interactions of age and dog ownership, age and weekday, province and gender, dog and poultry ownership, and dog ownership and weekday (**Table** **A**). Female participants living in Limburg, Flemish Brabant or West Flanders had more contacts than those living in Antwerp, but male participants living in Flemish Brabant had a smaller lower number of contacts compared to male participants living in Antwerp.

In modeling physical number of contacts, the overdispersion parameter was estimated at 1.71 (95% CI [1.56, 1.86]) yielding significant overdispersion. In addition, we also found significant two-way interactions of age and gender, age and weekday, province and gender, and animal ownership and weekday (**Table** **B**). After including cat, dog, livestock and poultry ownership separately, we found significant effects of household size, province and weekday, as well as significant two-way interactions of age and gender, age and weekday, and province and gender (**Table** **C**).

**Table** **A**: Weighted-negative binomial regression for the total number of contacts^†^ in Flanders, Belgium, 2010-2011 (n=1742)

| Covariate | Sample size | Parameter estimates (SE^§^) | RNC^§^ | 95% CI^§^ for RNC | P value |
| --- | --- | --- | --- | --- | --- |
| **Age** |  |  |  |  | **0.008** |
| 0-5 years^*^ | 174 |  | 1.00 |  |  |
| 6-11 years | 127 | 0.27 (0.24) | 1.31 | [0.82, 2.09] |  |
| 12-17 years | 79 | 0.18 (0.25) | 1.19 | [0.73, 1.96] |  |
| 18-44 years | 621 | 0.53 (0.18) | 1.70 | [1.19, 2.41] |  |
| 45-64 years | 466 | 0.59 (0.19) | 1.81 | [1.25, 2.60] |  |
| 65+ years | 275 | 0.25 (0.25) | 1.29 | [0.79, 2.10] |  |
| **Household size** |  |  |  |  | **<0.001** |
| 1^*^ | 98 |  | 1.00 |  |  |
| 2 | 312 | 0.15 (0.08) | 1.16 | [0.99, 1.34] |  |
| 3 | 328 | 0.22 (0.08) | 1.24 | [1.07, 1.44] |  |
| 4 | 439 | 0.29 (0.08) | 1.34 | [1.15, 1.55] |  |
| ≥5 | 218 | 0.39 (0.08) | 1.48 | [1.26, 1.73] |  |
| Missing | 347 | 0.03 (0.10) | 1.03 | [0.84, 1.26] |  |
| **Dog ownership** |  |  |  |  | **0.883** |
| Owner^*^ | 434 |  | 1.00 |  |  |
| Not owner | 1308 | -0.03 (0.18) | 0.97 | [0.68, 1.39] |  |
| **Poultry ownership** |  |  |  |  | **0.227** |
| Owner^*^ | 270 |  | 1.00 |  |  |
| Not owner | 1472 | 0.10 (0.08) | 1.10 | [0.94, 1.28] |  |
| **Gender** |  |  |  |  | **0.458** |
| Female^*^ | 930 |  | 1.00 |  |  |
| Male | 812 | 0.05 (0.06) | 1.05 | [0.92, 1.19] |  |
| **Province**^‡^ |  |  |  |  | **0.029** |
| Antwerp^*^ | 487 |  |  |  |  |
| Limburg | 264 | 0.18 (0.07) | 1.20 | [1.04, 1.39] |  |
| East Flanders | 407 | 0.06 (0.06) | 1.06 | [0.93, 1.20] |  |
| Flemish Brabant | 257 | 0.20 (0.07) | 1.22 | [1.05, 1.41] |  |
| West Flanders | 327 | 0.13 (0.07) | 1.13 | [0.99, 1.31] |  |
| **Weekday indicator** |  |  |  |  | **<0.001** |
| Weekend^*^ | 419 |  | 1.00 |  |  |
| Weekday | 1323 | 0.58 (0.16) | 1.79 | [1.30, 2.45] |  |
| **Holiday indicator** |  |  |  |  | **0.004** |
| Regular period^*^ | 1632 |  | 1.00 |  |  |
| Holiday period | 110 | -0.22 (0.07) | 0.81 | [0.70, 0.93] |  |
| **Age: Dog ownership** |  |  |  |  | **0.014** |
| 6-11 years: Not owner | 95 | 0.37 (0.21) | 1.45 | [0.96, 2.19] |  |
| 12-17 years: Not owner | 58 | 0.38 (0.22) | 1.47 | [0.96, 2.24] |  |
| 18-44 years: Not owner | 436 | 0.09 (0.16) | 1.09 | [0.79, 1.51] |  |
| 45-64 years: Not owner | 341 | -0.03 (0.17) | 0.97 | [0.69, 1.36] |  |
| 65+ years: Not owner | 243 | 0.39 (0.21) | 1.47 | [0.98, 2.21] |  |
| **Age: Weekday indicator** |  |  |  |  | **<0.001** |
| 6-11 years: Weekday | 96 | -0.28 (0.21) | 0.75 | [0.50, 1.13] |  |
| 12-17 years: Weekday | 58 | -0.20 (0.21) | 0.82 | [0.54, 1.24] |  |
| 18-44 years: Weekday | 466 | -0.71 (0.16) | 0.49 | [0.36, 0.67] |  |
| 45-64 years: Weekday | 358 | -0.75 (0.16) | 0.47 | [0.34, 0.65] |  |
| 65+ years: Weekday | 221 | -1.03 (0.18) | 0.36 | [0.25, 0.51] |  |
| **Province: Gender** |  |  |  |  | **0.027** |
| Limburg: Male | 124 | 0.03 (0.11) | 1.03 | [0.83, 1.28] |  |
| East Flanders: Male | 176 | -0.04 (0.10) | 0.96 | [0.79, 1.15] |  |
| Flemish-Brabant: Male | 121 | -0.32 (0.11) | 0.73 | [0.59, 0.90] |  |
| West Flanders: Male | 171 | -0.10 (0.10) | 0.91 | [0.74, 1.11] |  |
| **Dog ownership:**  **Poultry ownership** |  |  |  |  | **0.018** |
| Not onwer (dog): Not owner (poultry) | 1139 | -0.23 (0.10) | 0.79 | [0.66, 0.96] |  |
| **Dog ownership:**  **Weekday indicator** |  |  |  |  | **0.046** |
| Not owner: weekday | 998 | 0.19 (0.09) | 1.21 | [1.00, 1.45] |  |
| Dispersion=2.47 (SE=0.10), 95% CI [2.27, 2.66] | | | | | |

^*^Reference Category.

^†^We included cat, dog, livestock and poultry ownership as a covariate instead of animal ownership.

^‡^We excluded 14 observations with missing provinces because of not being enough for estimation.

^§^RNC=Relative Number of Contact, SE=Standard Error and CI=Confidence Interval.

**Table** **B**: Weighted-negative binomial regression for the physical number of contacts**^†^** in Flanders, Belgium, 2010-2011 (n=1742)

| Covariate | Sample size | Parameter estimates (SE**^§^**) | RNC**^§^** | 95% CI**^§^** for RNC | P-value |
| --- | --- | --- | --- | --- | --- |
| **Age** |  |  |  |  | **0.046** |
| 0-5 years^*^ | 174 |  | 1.00 |  |  |
| 6-11 years | 127 | 0.65 (0.24) | 1.91 | [1.19, 3.07] |  |
| 12-17 years | 79 | 0.15 (0.24) | 1.17 | [0.72, 1.88] |  |
| 18-44 years | 621 | 0.17 (0.19) | 1.19 | [0.82, 1.70] |  |
| 45-64 years | 466 | 0.06 (0.19) | 1.06 | [0.72, 1.55] |  |
| 65+ years | 275 | 0.33 (0.24) | 1.39 | [0.86, 2.23] |  |
| **Household size** |  |  |  |  | **<0.001** |
| 1^*^ | 98 |  | 1.00 |  |  |
| 2 | 312 | 0.08 (0.10) | 1.08 | [0.89, 1.31] |  |
| 3 | 328 | 0.16 (0.10) | 1.17 | [0.97, 1.41] |  |
| 4 | 439 | 0.28 (0.10) | 1.32 | [1.09, 1.59] |  |
| ≥5 | 218 | 0.42 (0.10) | 1.53 | [1.25, 1.86] |  |
| Missing | 347 | -0.01 (0.13) | 0.99 | [0.77, 1.29] |  |
| **Animal ownership** |  |  |  |  | **0.029** |
| Owner^*^ | 1042 |  | 1.00 |  |  |
| Not owner | 700 | -0.20 (0.09) | 0.82 | [0.68, 0.98] |  |
| **Gender** |  |  |  |  | **0.715** |
| Female^*^ | 930 |  | 1.00 |  |  |
| Male | 812 | 0.06 (0.17) | 1.06 | [0.76, 1.49] |  |
| **Province**^‡^ |  |  |  |  | **0.028** |
| Antwerp^*^ | 487 |  | 1.00 |  |  |
| Limburg | 264 | 0.11 (0.09) | 1.12 | [0.94, 1.34] |  |
| East Flanders | 407 | 0.13 (0.08) | 1.14 | [0.98, 1.33] |  |
| Flemish Brabant | 257 | 0.30 (0.09) | 1.35 | [1.13, 1.62] |  |
| West Flanders | 327 | 0.11 (0.09) | 1.11 | [0.93, 1.33] |  |
| **Weekday indicator** |  |  |  |  | **0.006** |
| Weekend^*^ | 419 |  | 1.00 |  |  |
| Weekday | 1323 | 0.51 (0.18) | 1.66 | [1.16, 2.34] |  |
| **Age: Gender** |  |  |  |  | **0.004** |
| 6-11 years: Male | 68 | -0.32 (0.22) | 0.73 | [0.47, 1.12] |  |
| 12-17 years: Male | 40 | 0.13 (0.22) | 1.14 | [0.74, 1.77] |  |
| 18-44 years: Male | 269 | 0.02 (0.17) | 1.02 | [0.73, 1.43] |  |
| 45-64 years: Male | 200 | 0.33 (0.18) | 1.39 | [0.98, 1.97] |  |
| 65+ years: Male | 131 | -0.02 (0.19) | 0.98 | [0.67, 1.43] |  |
| **Age: Weekday indicator** |  |  |  |  | **<0.001** |
| 6-11 years: Weekday | 96 | -0.39 (0.25) | 0.67 | [0.41, 1.10] |  |
| 12-17 years: Weekday | 58 | -0.34 (0.25) | 0.71 | [0.43, 1.17] |  |
| 18-44 years: Weekday | 466 | -0.76 (0.19) | 0.47 | [0.32, 0.68] |  |
| 45-64 years: Weekday | 358 | -0.88 (0.20) | 0.41 | [0.28, 0.61] |  |
| 65+ years: Weekday | 221 | -1.07 (0.22) | 0.34 | [0.22, 0.53] |  |
| **Province: Gender** |  |  |  |  | **0.026** |
| Limburg: Male | 124 | -0.05 (0.14) | 0.95 | [0.73, 1.25] |  |
| East Flanders: Male | 176 | -0.12 (0.12) | 0.88 | [0.70, 1.12] |  |
| Flemish-Brabant: Male | 121 | -0.43 (0.13) | 0.65 | [0.50, 0.85] |  |
| West Flanders: Male | 171 | -0.10 (0.13) | 0.91 | [0.71, 1.16] |  |
| **Animal ownership: Weekday indicator** |  |  |  |  | **0.025** |
| Not an owner: Weekday | 530 | 0.24 (0.10) | 1.27 | [1.03, 1.56] |  |
| Dispersion=1.71 (SE=0.08), 95% CI [1.56, 1.86] | | | | |  |

^*^Reference category.

**^†^**We included animal ownership as a covariate.

^‡^We excluded 14 observations with missing provinces because of sparseness.

**^§^**RNC=relative number of contact, SE=standard error and CI=confidence interval.

**Table** **C**: Weighted-negative binomial regression for the physical number of contacts^†^ in Flanders, Belgium, 2010-2011 (n=1742)

| Covariate | Sample size | Parameter estimates (SE^§^) | RNC^§^ | 95% CI^§^ for RNC | P value |
| --- | --- | --- | --- | --- | --- |
| **Age** |  |  |  |  | **0.070** |
| 0-5 years^*^ | 174 |  | 1.00 |  |  |
| 6-11 years | 127 | 0.64 (0.24) | 1.90 | [1.18, 3.06] |  |
| 12-17 years | 79 | 0.17 (0.24) | 1.18 | [0.73, 1.90] |  |
| 18-44 years | 621 | 0.18 (0.19) | 1.20 | [0.83, 1.72] |  |
| 45-64 years | 466 | 0.07 (0.19) | 1.07 | [0.73, 1.56] |  |
| 65+ years | 275 | 0.22 (0.24) | 1.24 | [0.77, 1.98] |  |
| **Household size** |  |  |  |  | **<0.001** |
| 1^*^ | 98 |  | 1.00 |  |  |
| 2 | 312 | 0.08 (0.10) | 1.08 | [0.89, 1.30] |  |
| 3 | 328 | 0.15 (0.09) | 1.17 | [0.97, 1.41] |  |
| 4 | 439 | 0.28 (0.09) | 1.32 | [1.10, 1.59] |  |
| ≥5 | 218 | 0.43 (0.10) | 1.54 | [1.26, 1.87] |  |
| Missing | 347 | -0.01 (0.13) | 0.99 | [0.77, 1.29] |  |
| **Gender** |  |  |  |  | **0.728** |
| Female^*^ | 930 |  | 1.00 |  |  |
| Male | 812 | 0.06 (0.17) | 1.06 | [0.76, 1.49] |  |
| **Province**^‡^ |  |  |  |  | **0.031** |
| Antwerp^*^ | 487 |  | 1.00 |  |  |
| Limburg | 264 | 0.11 (0.09) | 1.11 | [0.93, 1.34] |  |
| East Flanders | 407 | 0.14 (0.08) | 1.14 | [0.98, 1.34] |  |
| Flemish Brabant | 257 | 0.30 (0.09) | 1.35 | [1.12, 1.61] |  |
| West Flanders | 327 | 0.10 (0.09) | 1.11 | [0.93, 1.32] |  |
| **Weekday indicator** |  |  |  |  | **<0.001** |
| Weekend^*^ | 419 |  | 1.00 |  |  |
| Weekday | 1323 | 0.59 (0.17) | 1.81 | [1.28, 2.54] |  |
| **Age: Gender** |  |  |  |  | **0.004** |
| 6-11 years: Male | 68 | -0.31 (0.22) | 0.74 | [0.48, 1.13] |  |
| 12-17 years: Male | 40 | 0.13 (0.22) | 1.14 | [0.74, 1.77] |  |
| 18-44 years: Male | 269 | 0.02 (0.17) | 1.02 | [0.73, 1.44] |  |
| 45-64 years: Male | 200 | 0.34 (0.18) | 1.40 | [0.99, 1.99] |  |
| 65+ years: Male | 131 | -0.01 (0.19) | 0.99 | [0.68, 1.44] |  |
| **Age: Weekday indicator** |  |  |  |  | **<0.001** |
| 6-11 years: Weekday | 96 | -0.40 (0.25) | 0.67 | [0.41, 1.09] |  |
| 12-17 years: Weekday | 58 | -0.36 (0.25) | 0.70 | [0.42, 1.15] |  |
| 18-44 years: Weekday | 466 | -0.77 (0.19) | 0.46 | [0.32, 0.67] |  |
| 45-64 years: Weekday | 358 | -0.89 (0.20) | 0.41 | [0.28, 0.61] |  |
| 65+ years: Weekday | 221 | -0.96 (0.22) | 0.38 | [0.25, 0.59] |  |
| **Province: Gender** |  |  |  |  | **0.026** |
| Limburg: Male | 124 | -0.05 (0.14) | 0.95 | [0.73, 1.24] |  |
| East Flanders: Male | 176 | -0.14 (0.12) | 0.87 | [0.69, 1.10] |  |
| Flemish-Brabant: Male | 121 | -0.43 (0.13) | 0.65 | [0.50, 0.84] |  |
| West Flanders: Male | 171 | -0.10 (0.13) | 0.91 | [0.71, 1.16] |  |
| Dispersion= 1.71 (SE=0.08), 95% CI [1.56 1.85] | | | | | |

^*^Reference Category.

^†^We included cat, dog, livestock and poultry ownership as a covariate instead of animal ownership.

^‡^We excluded 14 observations with missing provinces because of not being enough for estimation.

^§^RNC=Relative Number of Contact, SE=Standard Error and CI=Confidence Interval.
