# Supplementary material for: Animal Ownership and Touching Enrich the Context of Social Contacts Relevant to the Spread of Human Infectious Diseases
Source: PLoS One. 2015 Jul 20;10(7):e0133461. doi: 10.1371/journal.pone.0133461 (PMC4508096; doi:10.1371/journal.pone.0133461)
Supplement: S5 File — (DOCX) [file pone.0133461.s008.docx]

## S5 File. Major outbreak probability of zoonoses with flu-like transmissibility, conditional on their presence in animal groups

According to the Belgium census data 2000, we categorized age (crucial demographic factor) into 15 categories: 0-4, 5-9, 10-14, 15-19, 20-24, 25-29, 30-34, 35-39, 40-44, 45-49, 50-54, 55-59, 60-64, 65-69 and 70+ years. Assuming pathogen spillover crossing the species barrier [***1***], we investigated the relative role of different zoonotic sources and exposed age groups in finding the probability of a human-transmissible pathogen will cause a major zoonotic outbreak as follows:

Let *PO_j_*=the probability that a human-transmissible pathogen will cause a major outbreak within a week in a particular age group *j*, conditional on the pathogen being present in pets, livestock and poultry, and being able to transmit to humans with a frequency that is proportional to the frequency of touching or owning these animals

where *Z_j_*= the probability that an infection will not die out given that it starts in age group *j*, *F_j_*= the proportion of people in age group *j* who are “exposed” to animals due to ownership or touching, and *P_j_*= the proportion of the *j^th^* age group in the population.

In order to compute *Z_j_*, we translated the continuous-time branching process from the context of demography to that of epidemiology [***2***], by approximating the random number of living individuals by the random number of infectious individuals. We assumed that a Poisson process could characterize the stochastic epidemic. The probability that an infection will die out, i.e. the extinction probability, characterized by the probability generating function denoted by *g(s)* is also called offspring distribution in branching processes.

Let *R_ij_*= the basic reproduction number to group *i* from group *j*, which is the expected number of infections generated by one primary case in age class *j* when introduced in a completely susceptible population of age class *i*

Since the branching processes are assumed independent, we have

For the branching process we can compute the probability of a minor outbreak, and for a major outbreak using the complementary probability. The extinction probability of the minor outbreak *s_i_*, is the smallest non-negative solution of *s_i_*=*g_i_(s)*. So, the probability that an infection will cause an epidemic given that it starts in-group *i*, *Z_i_*=*1-s_i_*.

Contact rates were used to calculate the transmission rates and R_ij_. The elements of the social contact matrix *m_ij_*, representing the mean number of contacts in age class *j* during one day reported by a respondent in age class *i*, can be estimated by the following expression, to which we refer as age-heterogeneous:

where *T_i_* is the number of participants in age class *i*, *w_t_^d^* is the diary weight for participant *t* and *y_ijt_* the reported number of contacts made by the participant *t* of age class *i* with someone of age class *j*. The reciprocal nature of contacts requires *m_ij_N_i_* to equal *m_ji_N_j_*, so we defined the elements of the social contact matrix *C*, representing the per capita daily contact rate between age classes, as:

where *N_i_* is the population size in age class *i*, obtained from demographic data (2000 Belgium census data). Finally, the next generation matrix R_ij_ is defined as:

where, *N_i_*=the population size in age class *i*, *D*=the mean duration of infectiousness (assumed to be 1 week), , *C*=contact matrix (a matrix with *c_ij_* as cells) and *q*=proportionality factor. The basic reproduction number *R_0_* can be calculated as the dominant eigenvalue of the next generation matrix. By way of example and without loss of generalisability we assumed a basic reproduction number of 1.5 [***3***] to estimate q. Note that the value of the basic reproduction number implies a certain level of transmissibility (which is in this case representative of (seasonal) influenza). Finally, the implicit expression given in equation 2 was solved for *s_j_* to determine *Z_j_* (i.e., *Z_j_= 1-s_j_*), for *i, j*: 1,2,…,15.

Assuming age-homogeneous contact patterns, we computed the overall average number of contacts (*m*) as follows:

where *n* stands for the number of participants in the observed data, ${w_{k}}^{d}$ is the diary weight for the *k^th^* participant, and $y_{k}$ is the reported number of contacts made by the *k^th^* participant with others.

Hence, the contact rates under the homogeneous assumption were computed by using *m* instead of *m_ij_* in equation 4. Under both assumptions, the proportion of people in age group *j* who are “exposed” to animals, *F_j_*, was calculated as the observed proportion of participants in each age group who owned and/or touched pets, livestock and poultry (see **Table A**).

**Table** **A**: The observed proportion of participants who owned/touched pets, livestock and poultry by age in Flanders, Belgium, 2010-2011

| Age group (in years) | Ownership (n=1756)* | | | Touching (n=1724)* | | |
| --- | --- | --- | --- | --- | --- | --- |
|  | Pets | Livestock | Poultry | Pets | Livestock | Poultry |
| 0-4 | 0.042 | 0.004 | 0.013 | 0.032 | 0.002 | 0.001 |
| 5-9 | 0.043 | 0.002 | 0.014 | 0.03 | 0.001 | 0.000 |
| 10-14 | 0.026 | 0.003 | 0.007 | 0.02 | 0.003 | 0.001 |
| 15-19 | 0.024 | 0.003 | 0.009 | 0.024 | 0.001 | 0.000 |
| 20-24 | 0.023 | 0.002 | 0.006 | 0.021 | 0.000 | 0.001 |
| 25-29 | 0.043 | 0.007 | 0.009 | 0.038 | 0.004 | 0.001 |
| 30-34 | 0.031 | 0.002 | 0.010 | 0.035 | 0.000 | 0.002 |
| 35-39 | 0.044 | 0.003 | 0.011 | 0.042 | 0.003 | 0.002 |
| 40-44 | 0.051 | 0.006 | 0.016 | 0.049 | 0.003 | 0.002 |
| 45-49 | 0.055 | 0.007 | 0.014 | 0.052 | 0.005 | 0.002 |
| 50-54 | 0.052 | 0.001 | 0.012 | 0.053 | 0.001 | 0.003 |
| 55-59 | 0.019 | 0.001 | 0.009 | 0.017 | 0.001 | 0.000 |
| 60-64 | 0.022 | 0.003 | 0.009 | 0.020 | 0.001 | 0.001 |
| 65-69 | 0.017 | 0.000 | 0.006 | 0.015 | 0.000 | 0.001 |
| 70+ | 0.024 | 0.001 | 0.011 | 0.024 | 0.001 | 0.005 |

^*^We excluded 12 (0.7%) and 44 (2.5%) missing observations via owning and touching, respectively.

**References**

1. Singh S, Schneider DJ, and Myers CR. The structure of infectious disease outbreaks across the animal-human interface; 2013. arXiv preprint. Available: arXiv: 1307.4628v1.
2. Diekmann O, Heesterbeek H and Britton T. Mathematical Tools for Understanding Infectious Disease Dynamics. Princeton University Press; 2012.
3. Flahaut A, Letrait S, Blin P, Hazout S, Ménarès J and Valleron AJ. Modelling the 1985 influenza epidemic in France. Stat Med. 1988;7: 1147-1155.
